# Discoveries Interview: Professor Roman Zubarev on the revolution in polypeptide sequencing

**DOI:** 10.15190/d.2015.39

**Published:** 2015-07-27

**Keywords:** professor RomanZubarev, interview

**Figure 1:**
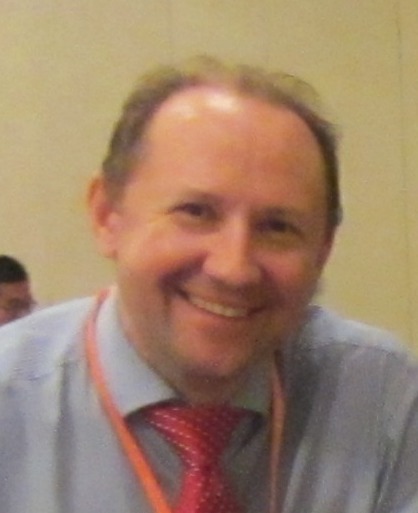
Professor Roman A. Zubarev

**Dr. Roman A. Zubarev** is a professor of medical proteomics in the Dept. of Medical Biochemistry and Biophysics at the Karolinska Institutet, Sweden.

Prof. Zubarev is known for the development of the fragmentation technique of electron-capture dissociation, while in Fred McLafferty’s lab at Cornell University.

Professor Zubarev did his Master in applied physics at Moscow Engineering Physics Institute in 1986 and his PhD in ion physics at Uppsala University, Sweden, in 1997, investigating desorption of bioorganic ions from surfaces. His main research interests include the mechanisms of Alzheimer’s disease and cancer, reversibility in biology and biological effects of stable isotopes. He published over 200 publications and has several patents. He is a member of Human Proteome Organisation (HUPO), of a number of national mass spectrometry societies, and of editorial boards of several journals in proteomics and mass spectrometry. Professor Zubarev’s major awards include: Curt Brunnée Award “for outstanding contribution to the development of mass spectrometry instrumentation” (2006) and Klaus Biemann Medal for “a significant achievement in basic or applied mass spectrometry made by an individual early in his or her career” (2007).

## 1. Can you define in simple words why polypeptide sequencing is important?

Polypeptides, including proteins and functional peptides, create basis for life; they perform vast amount of work in the cell and organism. Polypeptides are a string made of 20 types of amino acid residues; these types are common for all polypeptides from all biological organisms. The difference between any two polypeptides is in the order of the amino acid residues, i.e. the sequence, as well is in the chemical alterations of these residues called posttranslational modifications (PTMs). The polypeptide sequence is a text written by nature using amino acid residues as letters and PTMs as punctuation marks. Reading this text, that is performing sequencing, is the first necessary step towards understanding the meaning of that text.

In principle, genetics provides sequences of DNAs or RNAs that code for polypeptide sequences, but these nucleic acid sequences are just blueprints for the amino acid sequences found in polypeptides. In the process of polypeptide expression, these sequences can be altered (so called pre-translational modification). Besides, PTMs are not coded on nucleic acid level. Therefore, there is no real substitution for sequencing the actual polypeptides.

## 2. How polypeptide sequencing methods evolved over the time?

Sequencing in early 1950s by biochemical methods of the two polypeptide chains of bovine insulin containing altogether 51 amino acid residues took years to complete and deserved a Nobel prize. Later, polypeptide sequencing was automated through the Edman degradation process, which still used wet chemistry and chromatography at every step, and took on average one hour per residue. The first attempts to sequence polypeptides by mass spectrometry (MS) were made in 1950s, but it is with the invention of so-called “soft ionization” methods (2002 Nobel prize in Chemistry) that the MS-based methods became fast (milliseconds) and routine. In that approach, polypeptides are first ionized by adding to them one or several charges, mostly in form of protons. Simultaneously, the newly formed polypeptide ions enter the gas phase, which is actually a rather good vacuum of a mass spectrometer. There they are heated by collisions with neutral gas, and dissociate into fragments. Mass spectrometer measures the masses of these fragments. Then the software, knowing the rules of fragmentation, determines the order of amino acids in the original sequence.

## 3. What is electron-capture dissociation (ECD)?

ECD is an alternative method to fragment polypeptides in the gas phase. Instead of collisions with a neutral gas, the polypeptide ions collide in ECD with low-energy electrons^1,2^. Coulombic attraction between the positive polypeptide ions and negative electrons leads to electron capture by the former, while recombination of a proton and the captured electron releases energy and creates unstable polypeptide radical species. These species very rapidly fall apart, creating fragments. Unlike the collision-induced dissociation that ruptures the peptide bond C-N, ECD ruptures the “next over” N-C_a_ bond, creating different fragments. Together, ECD and the conventional dissociation method produce sequencing data that are far more extensive and reliable than any of these two methods alone.

## 4. How your discovery of electron-capture dissociation helps understand and target human diseases?

ECD-based sequencing of polypeptides has many applications in life sciences. One of the most exciting emerging applications is sequencing the repertoire of human antibodies and linking them to disease. The old paradigm in immunology is that, because of the vast number of possible antibody sequences (the estimates vary between 10^13^ and 10^15^), no two humans would have exactly the same antibodies in blood. Recent research proved this paradigm to be untrue, especially in certain diseases. The presence in human organism of specific antigens, such as bacterial or viral infections, abnormal protein aggregates and so on, gives rise to antibodies that have a lot of sequence similarity in different people suffering from the same condition. Exploring this similarity will allow us not only to diagnose these diseases more accurately, but also identify more correctly the cause of these diseases. Both these steps are important for curing the diseases.

## 5. What will the field look like in 5-10 years?

One of the biggest problems mass spectrometry faces in sequencing human proteins is the great range of concentrations at which they appear in human cells (ca. 1:10^7^) and blood (ca. 1:10^11^). This is far greater than the dynamic range of the signal a mass spectrometer can handle, ca. 1:10^5^. Because of such a mismatch, extensive protein fractionation and enrichment is required, which slows down the analysis and makes it quite expensive. We expect that the next 5-10 years will see the dynamic range of instrumentation gradually approaching that of the protein samples, which will allow us to perform analysis “in one go”, very quickly and cheaply. We also hope that, by that time, most abundant and common antibody variants in human blood will be sequenced, and their relation to different diseases and health conditions will be established. This effort may result in rewriting a few medical textbooks, because some causes of common diseases may turn out to be different than generally assumed.

## 6. What advices do you have for young scientists?

Many new discoveries are practically begging to be made, and require no fancy instrumentation or extensive funding, just a fresh look at the problem.

The history of the ECD discovery is a case in point. Scientists have been working with free electrons for about a century, and with multiply charged polypeptides for a decade, and yet no-one before my former supervisor professor Fred W. McLafferty from Cornell has had an insight to merge these two species together. Once this was done in a proper way (which was my job), things became very easy. In hindsight, it is clear that fragmentation is exactly what should happen when unstable radicals form. Having said that, the type of fragmentation and its mechanism was difficult to predict.

Therefore, the advices for young scientists are the following: think of a novel experiment in your area and do it. If it fails, think how to improve it, and do it again. Also, look for unusual outcomes in conventional experiments, and investigate their causes to the bottom. Don’t discard any strange result just because it deviates from your expectations; instead, try to understand what exactly nature tries to tell us. Nature can’t hold its secrets very well and constantly reveals them to us. Unfortunately, our eyes are often trained to ignore new information, looking only for known and expected outcomes.

## 7. In your opinion, what are the most challenging, promising and/or the most rewarding areas of research?

I would name two exciting areas: reversibility and complexity.

Being trained as a physicist, I happen to believe that all processes should be reversible in time. This is because Quantum mechanics is the basis for everything, and Schrödinger’s equation underlying Quantum mechanics is symmetric in respect to time reversal. As a general principle this, however, cannot be demonstrated otherwise than through a series of experiments, in which the processes thought to be irreversible are reversed. In my lab we are working on such experiments. One focal point of efforts is the central dogma of molecular biology, first stated by Francis Crick in 1956 and then restated in 1970. It postulates, among other things, that protein translation is irreversible: there is no natural process that starts with protein sequence and ends up with a corresponding RNA or DNA sequence. We ask: why not? And look for such a process in vivo, as well as try to design it in a test tube. If one manages to do this, “PCR for proteins” may become more than a joke. Reversing other key biological processes, e.g. protein degradation through deamidation, may stop ageing and certain common diseases, such as Alzheimer’s. As for complexity, it should be more than a general term with fuzzy meaning. It is about time that science gave precise definition to complexity, assigned units to it and started to measure it quantitatively. The situation with complexity is similar to that with energy 100 years ago. In 1900, Poincaré grieved that we cannot give a general definition of energy, and thus the energy conservation law remains an important principle without experimental verification. This deficiency was later fixed through the work of brilliant theoretical physicists of the first decades of 20^th^ century. It is now time we do the same with complexity. Accurate measurements of complexity changes in an isolated system are likely to reveal that it remains conserved, similar to energy. It this is true, the complexity conservation law may revolutionize science and technology as much as did the energy conservation law.

Symmetry is perhaps the most important aspect reducing the complexity of a system. What emerged from our research on biological effects of stable isotopes is that more complex systems appear to be slower than more symmetric, and thus less complex, systems. If this is a general law, or at least an important rule, than modulating system’s complexity through its isotopic composition may cause arbitrary acceleration or deceleration of its growth and development, with almost infinite applications in physics, chemistry, biology and medicine.

Both of the above areas are very fundamental in nature, but advances in them can be made through relatively simple experiments that do not demand much resources, although do require a great deal of new thinking and ingenuity. I like to say that, at least in my lab, we are more limited by the shortage of new great ideas than by the lack of funding for pursuing them.

Opening one’s mind is never easy; on the contrary, it can be quite painful, as one definitely moves out of one’s comfort zone. Yet, great discoveries in science have been made in the past by the individuals who didn’t mind to take such a pain.
